# The risk of ischemic stroke and hemorrhagic stroke in Chinese adults with low-density lipoprotein cholesterol concentrations < 70 mg/dL

**DOI:** 10.1186/s12916-021-02014-4

**Published:** 2021-06-16

**Authors:** Zhijun Wu, Zhe Huang, Alice H. Lichtenstein, Yesong Liu, Shuohua Chen, Yao Jin, Muzi Na, Le Bao, Shouling Wu, Xiang Gao

**Affiliations:** 1 Department of Cardiology, Ruijin Hospital, Shanghai Jiaotong University School of Medicine, Shanghai, People’s Republic of China; 2 Department of Cardiology, Kailuan General Hospital, 57 Xinhua East Rd, Tangshan, 063000 People’s Republic of China; 3 Cardiovascular Nutrition Laboratory, JM USDA Human Nutrition Research Center on Aging, Tufts University, Boston, MA USA; 4 Department of Neurology, Kailuan General Hospital, Tangshan, People’s Republic of China; 5 Health Care Center, Kailuan Medical Group, Tangshan, People’s Republic of China; 6 Department of Nutritional Sciences, The Pennsylvania State University, State College, PA USA; 7 Department of Statistics, The Pennsylvania State University, 109 Chandlee Lab, State College, University Park, PA 16802 USA

**Keywords:** Low-density lipoprotein cholesterol, Stroke, Metabolic diseases, Machine learning, Conditional inference tree

## Abstract

**Background:**

The risk of stroke in individuals with very low low-density lipoprotein cholesterol (LDL-C) concentrations remains high. We sought to prioritize predictive risk factors for stroke in Chinese participants with LDL-C concentrations < 70 mg/dL using a survival conditional inference tree, a machine learning method.

**Methods:**

The training dataset included 9327 individuals with LDL-C concentrations < 70 mg/dL who were free of cardiovascular diseases and did not use lipid-modifying drugs from the Kailuan I study (N = 101,510). We examined the validity of this algorithm in a second Chinese cohort of 1753 participants with LDL-C concentrations < 70 mg/dL from the Kailuan II study (N = 35,856).

**Results:**

During a mean 8.5–9.0-year follow-up period, we identified 388 ischemic stroke cases and 145 hemorrhagic stroke cases in the training dataset and 20 ischemic stroke cases and 8 hemorrhagic stroke cases in the validation dataset. Of 15 examined predictors, poorly controlled blood pressure and very low LDL-C concentrations (≤ 40 mg/dL) were the top hierarchical predictors of both ischemic stroke risk and hemorrhagic stroke risk. The groups, characterized by the presence of 2–3 of aforementioned risk factors, were associated with a higher risk of ischemic stroke (hazard ratio (HR) 7.03; 95% confidence interval (CI) 5.01–9.85 in the training dataset; HR 4.68, 95%CI 1.58–13.9 in the validation dataset) and hemorrhagic stroke (HR 3.94, 95%CI 2.54–6.11 in the training dataset; HR 4.73, 95%CI 0.81–27.6 in the validation dataset), relative to the lowest risk groups (presence of 0–1 of these factors). There was a linear association between cumulative average LDL-C concentrations and stroke risk. LDL-C concentrations ≤ 40 mg/dL was significantly associated with increased risk of ischemic stroke (HR 2.07, 95%CI 1.53, 2.80) and hemorrhagic stroke (HR 2.70, 95%CI 1.70, 4.30) compared to LDL-C concentrations of 55–70 mg/dL, after adjustment for age, hypertension status, and other covariates.

**Conclusion:**

Individuals with extremely low LDL-C concentrations without previous lipid-modifying treatment could still be at high stroke risk.

**Trial registration:**

Chinese Clinical Trial Register, ChiCTR-TNRC-11001489. Registered on 24-08-2011.

**Supplementary Information:**

The online version contains supplementary material available at 10.1186/s12916-021-02014-4.

## Background

Low-density lipoprotein cholesterol (LDL-C) has been long regarded as one of the major pathogenic risk factors that increase the risk of cardiovascular diseases (CVD) and cerebrovascular diseases [1]. LDL-C lowering therapy has been demonstrated to be effective in reducing atherosclerotic disease risk substantially [2]. The LDL-C concentration of 70 mg/dL was considered as an appropriate target goal for optimal lipid management in people who are at high risk of CVD [3–5]. However, emerging observational evidence, suggested that the risk of ischemic stroke [6, 7] and hemorrhagic stroke [8, 9] remained high among those who had low concentrations of LDL-C. Little is known about the effect of longer-term habitual cumulative exposure to very low LDL-C concentrations (e.g., < 40 mg/dL).

It is thus of clinical significance to understand the factors related to the risk of cerebrovascular diseases in the population with low LDL-C concentrations. Whether other metabolic abnormalities (e.g., hypertension, diabetes mellitus, and obesity) contribute to the risk of stroke within the context of a low LDL-C concentration remains unclear. Important to consider, LDL-C concentrations and metabolic and lifestyle risk factors covary and may together have a synergistic or antagonistic effect on stroke-related outcomes.

Recently, machine learning techniques have been widely used for developing risk stratification algorithms due to their intuitive graphical representation [10]. Conditional inference tree is a fundamental machine learning method that recursively partitions participants into the homogenous group with similar outcome probabilities [11], to identify variable importance in the context of high-dimensional interactions [12]. We thus sought to prioritize the strong predictive risk factors of ischemic stroke risk and hemorrhagic stroke risk using a survival conditional inference tree (SCTREE) in a community-based cohort including 9327 participants with LDL-C concentrations < 70 mg/dL during an 8.5–9.0-year follow-up period. We further validated our findings in another independent cohort including 1753 participants with LDL-C concentrations < 70 mg/dL.

## Methods

### Study populations

We analyzed data from two independent ongoing cohorts—the Kailuan I study was used as the training dataset to develop the risk stratification algorithm, and the Kailuan II study was used as the validation dataset. The study design of these two cohorts has been described in detail previously [13, 14]. Briefly, both cohorts have been conducted in 11 hospitals affiliated with the Kailuan community in Tangshan city, China. The Kailuan I study was initiated in 2006–2007 and consisted of 101,510 Chinese adults (81,110 men and 20,400 women) aged 18 years or older living in the Kailuan community in 2006. The Kailuan II study was initiated in 2008–2010, including 35,856 adults, who lived in the Kailuan community but did not participate in the Kailuan I study. In both cohorts, participants who completed a questionnaire on demographic details, lifestyle behaviors (e.g., smoking and drinking habits), medication history, and medical comorbidity and underwent clinical and laboratory examinations at baseline and were followed every 2 years with the same strategy to update their health/lifestyle status. Included were 9327 participants in the training dataset and 1753 participants in the validation dataset based on the following criteria: (1) baseline LDL-C concentrations < 70 mg/dL, (2) cumulative average LDL-C concentrations < 70 mg/dL during the follow-up period (mean follow duration 9.0 and 8.5 years in the Kailuan I and II studies, respectively), (3) without CVD or cancer in or prior to the baseline, and (4) without lipid-modifying drugs at baseline or during the follow-up period (Supplementary Fig. 1).

### Assessment of outcomes (incident cases of ischemic stroke and hemorrhagic stroke)

The primary outcome was the first occurrence of ischemic stroke and hemorrhagic stroke. As previously described [9, 15, 16], all potential fatal and non-fatal cerebrovascular diseases cases were identified by the relevant International Classification of Diseases (ICD)-10th Revision [17, 18] from the Municipal Social Insurance Institution (covering all study participants) and the Hospital Discharge Register data and self-report questionnaires during the biennial follow-up surveys. Medical records for all the potential stroke cases were reviewed by 3 cardiologists and neurologists served at a committee of experts. The mortality information was obtained from Hebei Provincial Vital Statistics Offices or directly contacting the participants’ family members. Study clinicians reviewed death certificates and coded the main cause of death according to the ICD-10. Ischemic stroke and hemorrhagic stroke were defined as a neurological deficit of cerebrovascular cause that lasted more than 24 h or a significant lesion detected by computed tomography or magnetic resonance imaging [19].

### Assessment of potential predictors

Potential predictors include age, sex, smoking, alcohol intake, physical activity, body mass index, estimated glomerular filtration rate, urine protein, high-sensitivity C-reactive protein, lipid profiles, heart rate, blood pressure, and blood glucose control status (Supplementary Table 1). To take advantage of biennially repeated assessment of predictors, we used cumulative average values of LDL-C concentrations and other continuous variables calculated from all available measures since the baseline survey, as previously described [9, 13, 20]. For instance, the average of 2006 and 2008 LDL-C concentrations was used to predict stroke events occurring during 2008–2010; and the average of 2006, 2008, and 2010 measures was used to predict stroke occurring during 2010–2012. This approach allowed us to reduce random within-person variation and capture the long-term effects of studied stroke risk factors.

Information on demographic data, lifestyle factors, and use of medications (e.g*.*, hypoglycemic agents and antihypertensives) was collected using a structured questionnaire [21]. Fasting (8–12 h) blood samples and random midstream morning urinary samples were collected at baseline and biennial face-to-fact interview and analyzed in the Central Laboratory of Kailuan General Hospital every 2 years. Serum concentrations of 6 traits (LDL-C, high-density lipoprotein cholesterol, triglyceride, glucose, high-sensitivity C-reactive protein, and creatinine) were measured by an auto-analyzer (Hitachi 747; Hitachi, Tokyo, Japan) using commercially available kits, as previously described [13, 20, 21]. The intra-assay and the inter-assay coefficient variation of all the traits were less than 10%. Proteinuria status was assessed using a dry-chemistry method and standard urinary sediment examination within 2 h (H12-MA test strips; Changchun Dirui Medical Technology Co., Ltd., Changchun, China) and measured by a urine analyzer (N-600; Changchun Dirui Medical Technology Co., Ltd.). The results were semi-quantified as negative (< 15 mg/dL), trace (15–29 mg/dL), 1+ (30–300 mg/dL), 2+ (300–1000 mg/dL), or 3+ (> 1000 mg/dL) [22]. The estimated glomerular filtration rate (eGFR) was calculated according to the Chronic Kidney Disease Epidemiology Collaboration equation considering creatinine, sex, and age [23].

Weight and height were measured by trained field workers (nurses and physicians) during the survey, and body mass index was calculated as weight (kg)/height (m^2^).

Systolic blood pressure (SBP) and diastolic blood pressure (DBP) were measured twice from the seated position using a mercury sphygmomanometer, and the mean of the two readings was used for the analyses [14, 24]. Blood pressure control status was classified as follows: (1) well-controlled, SBP < 140 mmHg and DBP < 90 mmHg without treatment; (2) well-controlled, SBP < 140 mmHg and DBP < 90 mmHg with certain or uncertain information on drugs; (3) poorly controlled, SBP ≥ 140 mmHg or DBP ≥ 90 mmHg without treatment; and (4) poorly controlled, SBP ≥ 140 mmHg or DBP ≥ 90 mmHg with certain or uncertain information on drugs. Heart rate was detected by electrocardiogram at baseline and during follow-up surveys, as described previously [13, 24].

Given a long-term effect of high hyperglycemia on cerebrovascular disease occurrence, we classified all participants into 4 categories according to their glycemic control levels: (1) well-controlled, fasting blood glucose (FBG) < 126 mg/dL without administration of glucose-lowering drugs; (2) well-controlled, FBG < 126 mg/dL with certain or uncertain information on drugs; (3) poorly controlled, FBG ≥ 126 mg/dL without treatment; and (4) poorly controlled, FBG ≥ 126 mg/dL with certain or uncertain information on drugs (Supplementary Table 1).

### Statistical analysis

The person-time for each participant was calculated from the date of the baseline survey to the date of any stroke event diagnosis, lost to follow-up due to migrations or other reason (8.53%), mortality, or the end of follow-up, 31 December 2016, whichever came first.

The SCTREE model was used to develop a risk stratification algorithm for stroke risk in the Kailuan I study with 15 candidate attributes (Supplementary Table 1). SCTREE recursively partitions the dataset into smaller subsets for selecting the top predictor and corresponding cutoff value with the largest weighted Kaplan-Meier estimate. The incidence of stroke cases and survival time within each terminal node were calculated to generate an associated risk stratification tree.

The hazard ratios (HRs) and 95% confidence intervals (CIs) were calculated to compare the stroke risk across the risk groups generated by SCTREE. The performance of the SCTREE-developed risk stratification algorithm was further evaluated and compared using the validation dataset (the Kailuan II study).

We also conducted multivariate Cox regression and propensity score-matched Cox regression including the same predictors that were identified by the SCTREE analysis. The predictive ability and accuracy of the SCTREE and multivariate Cox models were compared using area under the receiver operating characteristic curves (AUC) [25] and Brier score [26]. A higher AUC with a lower Brier score was considered as a better prediction performance.

A Cox proportional hazards model was used to assess the association between the cumulative average values of LDL-C according to predefined groups with clinically meaningful cutoffs (≤ 40 mg/dL, 40–55 mg/dL, and 55–70 mg/dL) [27] and quartiles, and stroke risk. We also conducted other sensitivity analyses by excluding participants with eGFR < 60 ml/min/1.73 m^2^ or a 10-year Framingham risk score > 30% [28]. Considering malnutrition, as suggested by low BMI, which was associated with low concentrations of LDL-C and increased risk of stroke, we performed a sensitivity analysis by excluding participants with BMI < 18.5 kg/m^2^ [29]. To remove the confounding effect of treatment of hypertension or diabetes mellitus, we further performed sensitivity analyses by excluding those who used blood pressure-lowering drugs or glucose-lowering drugs.

We used the random survival forests algorithm on all 15 variables to validate the risk precision of the SCTREE model. The variables at a higher rank had a smaller minimal depth of a maximal subtree (a shorter distance from the root node to the parent node of the closest maximal subtree). We extracted the variable importance (VIMP) of each individual predictor to reflect the predictive abilities of the variables identified by the random survival forests algorithm [30]. Since VIMP is the increase of prediction errors after permuting the variable under consideration, a positive VIMP value indicates the variable improves the prediction accuracy, and a negative VIMP value indicates the variable leads to overfitting [31].

All statistical analyses were conducted using the R version 3.6.3 software (R Foundation for Statistical Computing, Vienna, Austria) and STATA12.0 (Stata Corporation, College Station, TX, USA). All statistical tests were 2-sided with a P value < 0.05 regarded as significant.

## Results

The cumulative average LDL-C concentrations of the training and validation datasets were similar. In contrast, participants in the validation cohort were younger and had a higher proportion of women, smoker, and drinker; low BMI; high level of heart rate and eGFR; high concentrations of triglyceride and LDL-C; and low concentrations of high-density lipoprotein cholesterol and engaged in a low level of physical activity and well-controlled blood pressure and blood glucose (Table 1). Identified were 388 ischemic stroke cases and 145 hemorrhagic stroke cases in the Kailuan I study with a mean of 9.0 years of follow-up and 20 ischemic stroke cases and 8 hemorrhagic stroke in the Kailuan II study with a mean of 8.5 years of follow-up.

**Table 1:** Baseline characteristics of Kailuan I study (training dataset) and Kailuan II study (validation dataset) participants with low-density lipoprotein cholesterol concentrations < 70 mg/dL

	Training dataset (Kailuan I study)	Validation dataset (Kailuan II study)
Number	9327	1753
Age, years	57.3 ± 13.7^a^	43.6 ± 15.1
Men, %	78.8	70.3
BMI, kg/m^2^	24.2 ± 3.39	23.8 ± 3.51
TG, mg/dL	144.4 ± 144.1	170.3 ± 186.5
LDL-C, mg/dL	55.6 ± 12.6	57.4 ± 10.4
HDL-C, mg/dL	58.5 ± 17.5	53.4 ± 15.7
HR, bpm	73.6 ± 9.48	74.1 ± 8.80
eGFR, ml/min/1.73 m^2^	88.4 ± 19.0	97.8 ± 21.9
hs-CRP, mg/L^b^	0.48 ± 1.29	0.47 ± 1.04
Urine protein		
Negative	89.8	88.8
Trace	6.28	3.04
+	2.38	6.55
++	1.05	1.17
+++	0.54	0.47
Physical activity, %		
Inactive	5.41	24.1
Moderately active	81.7	60.0
Vigorously active	12.9	15.9
Current smoker, %	28.8	39.5
Current drinker, %	31.1	39.4
Blood pressure control, %		
Well-controlled	63.9	70.4
Poorly controlled	36.1	29.6
Blood glucose control, %		
Well-controlled	91.8	92.7
Poorly controlled	8.26	7.26

*Abbreviations*: *BMI*, body mass index; *TG*, triglyceride; *LDL-C*, low-density lipoprotein cholesterol; *HDL-C*, high-density lipoprotein cholesterol; *HR*, heart rate; *eGFR*, estimated glomerular filtration rate; *hs-CRP*, high-sensitivity C-reactive protein

^a^Mean ± standard deviation

^b^hs-CRP was log-transformed

**Fig. 1:**
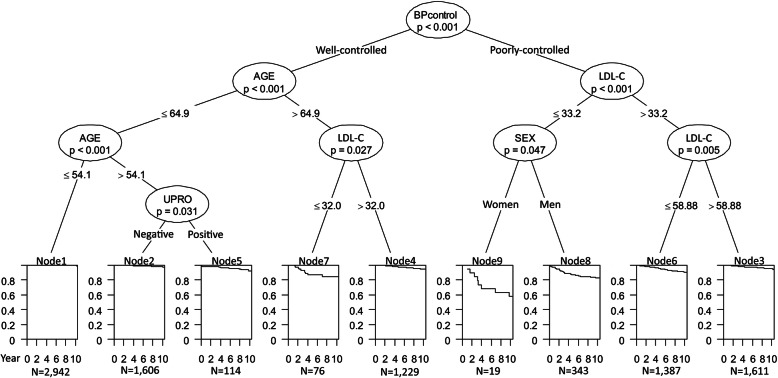
Conditional inference tree for ischemic stroke in individuals with low-density lipoprotein cholesterol concentrations < 70 mg/dL in the Kailuan I study. The terminal nodes show the Kaplan-Meier curves. BP, blood pressure; LDL-C, low-density lipoprotein cholesterol; UPRO, urine protein

Of the 15 variables that were examined, the first risk factor identified was blood pressure control status, followed by age and LDL-C concentrations for ischemic stroke. Participants (i) with poorly controlled blood pressure and LDL-C concentrations ≤33.2 mg/dL and (ii) with well-controlled blood pressure, aged > 64.9 years, and LDL-C concentrations ≤32.0 mg/dL had the highest ischemic stroke risk among the 9 sub-groups identified by the SCTREE model (Fig. 1). The HRs for these high-risk sub-groups, compared with the sub-group with the lowest stroke risk (well-controlled blood pressure and age ≤ 54.1 years), were more than 20 (P < 0.001 for all) (Table 2).

**Table 2:** Adjusted hazards ratios and 95% confidence intervals for the risk of ischemic stroke and hemorrhagic stroke across terminal nodes in the Kailuan I participants with low-density lipoprotein cholesterol concentrations < 70 mg/dL

	Ischemic stroke, HR (95%CI)								
	Node1	Node2	Node3	Node4	Node5	Node6	Node7	Node8	Node9
Case/person-years	15/28,515	37/15,483	78/14,743	60/10,818	8/1026	119/12,021	10/547	53/2553	8/143
LDL-C ± SD, mg/dL	56.8 ± 10.9	57.3 ± 10.8	64.6 ± 3.03	57.7 ± 8.76	55.6 ± 11.9	50.1 ± 6.48	19.2 ± 8.90	20.3 ± 8.53	20.6 ± 9.63
Multivariate model^a^	1.00 (reference)	3.73 (2.01, 6.92)	7.07 (3.98, 12.6)	7.38 (4.05, 13.4)	8.44 (3.04, 23.4)	13.0 (7.41, 22.7)	20.8 (7.86, 54.8)	23.1 (12.4, 43.1)	76.3 (30.2, 192.7)
Excluding eGFR < 60 ml/min/1.73 m^2^	1.00 (reference)	3.60 (1.94, 6.70)	6.06 (3.37, 10.9)	7.10 (3.85, 13.1)	8.86 (3.19, 24.6)	12.8 (7.30, 22.4)	18.9 (6.69, 53.4)	24.6 (13.1, 46.2)	62.2 (23.3, 166.3)
Excluding Framingham risk score > 30%	1.00 (reference)	3.82 (2.02, 7.23)	6.41 (3.38, 12.1)	6.71 (3.29, 13.7)	10.4 (3.74, 29.2)	12.3 (6.74, 22.4)	24.2 (6.74, 86.6)	23.3 (11.2, 48.1)	76.8 (24.7, 238.3)
Excluding BMI < 18.5 kg/m^2^	1.00 (reference)	3.75 (2.02, 6.94)	7.01 (3.94, 12.5)	7.51 (4.12, 13.7)	8.37 (3.02, 23.2)	13.0 (7.45, 22.8)	21.9 (8.29, 57.9)	23.1 (12.4, 43.1)	74.5 (29.5, 188.2)
Excluding blood pressure-lowering drugs	1.00 (reference)	3.48 (1.85, 6.56)	6.49 (3.50, 12.0)	7.24 (3.88, 13.5)	9.26 (3.32, 25.8)	11.0 (6.10, 19.8)	22.0 (8.24, 59.0)	22.3 (11.5, 43.2)	52.1 (16.8, 161.5)
Excluding glucose-lowering drugs	1.00 (reference)	3.69 (1.98, 6.87)	6.92 (3.87, 12.4)	7.81 (4.27, 14.3)	9.35 (3.36, 26.0)	13.1 (7.49, 23.0)	18.0 (6.39, 50.7)	21.8 (11.6, 40.9)	63.1 (23.7, 168.0)
	**Hemorrhagic stroke, HR (95%CI)**								
	**Node1**	**Node2**		**Node3**	**Node4**		**Node5**	**Node6**	
Case/person-years	13/41,704	13/4435		19/10,553	75/27,377		12/2102	13/739	
LDL-C ± SD, mg/dL	59.3 ± 7.25	28.9 ± 9.75		58.8 ± 7.51	57.8 ± 8.82		19.9 ± 8.60	20.2 ± 8.03	
Multivariate model^a^	1.00 (reference)	4.40 (2.07, 9.33)		6.59 (3.55, 12.2)	5.41 (2.22, 13.2)		9.74 (3.84, 24.7)	41.7 (17.2, 101.6)	
Excluding eGFR < 60 ml/min/1.73 m^2^	1.00 (reference)	4.58 (2.13, 9.85)		6.55 (3.52, 12.2)	4.68 (1.84, 11.9)		9.98 (3.78, 26.4)	37.5 (15.3, 91.6)	
Excluding Framingham risk score > 30%	1.00 (reference)	5.09 (2.02, 12.8)		5.01 (2.51, 9.99)	4.11 (1.52, 11.1)		10.7 (3.25, 35.2)	21.3 (6.86, 65.9)	
Excluding BMI < 18.5 kg/m^2^	1.00 (reference)	4.52 (2.13, 9.59)		6.46 (3.48, 12.0)	4.85 (1.91, 12.3)		9.71 (3.82, 24.7)	42.6 (17.4, 104.2)	
Excluding blood pressure-lowering drugs	1.00 (reference)	3.55 (1.57, 8.03)		5.39 (2.78, 10.4)	5.03 (2.05, 12.4)		7.73 (2.60, 23.1)	34.0 (12.8, 89.9)	
Excluding glucose-lowering drugs	1.00 (reference)	4.56 (2.14, 9.69)		6.42 (3.44, 12.0)	5.52 (2.26, 13.5)		10.2 (4.00, 25.8)	38.1 (15.2, 95.4)	

*Abbreviations*: *HR*, hazard ratio; *CI*, confidence interval; *LDL-C*, low-density lipoprotein cholesterol; *SD*, standard deviation; *eGFR*, estimated glomerular filtration rate; *BMI*, body mass index

^a^All models were adjusted for sex (men or women), age (year), physical activity (inactive, moderately active, or vigorously active), smoking and drinking status (never, former, occasional or daily), blood pressure status during follow-up (well-controlled or poorly controlled), blood glucose status during follow-up (well-controlled or poorly controlled), body mass index (kg/m^2^), urine protein (negative, trace, +, ++, or +++), heart rate (bpm), triglyceride (mg/dL), high-density lipoprotein cholesterol (mg/dL), low-density lipoprotein cholesterol (mg/dL), estimated glomerular filtration rate (ml/min/1.73 m^2^), and high-sensitivity C-reactive protein (mg/L)

**Fig. 2:**
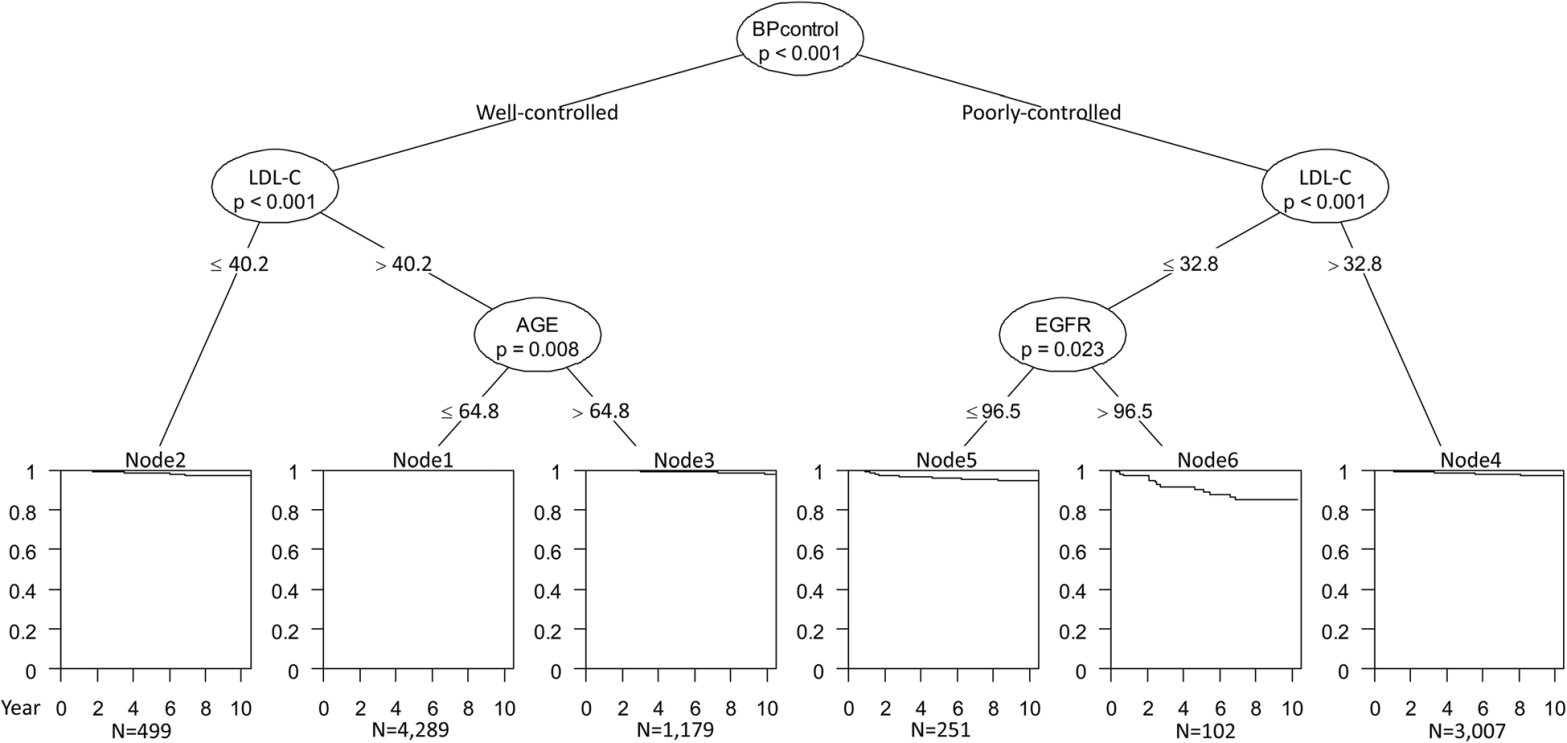
Conditional inference tree for hemorrhagic stroke in individuals with low-density lipoprotein cholesterol concentrations < 70 mg/dL in the Kailuan I study. The terminal nodes show the Kaplan-Meier curves. BP, blood pressure; LDL-C, low-density lipoprotein cholesterol; eGFR, estimated glomerular filtration rate

Poorly controlled blood pressure and low LDL-C concentrations were identified as the main top discriminators for hemorrhagic stroke (Fig. 2 and Supplementary Table 2). Participants with poorly controlled blood pressure and LDL-C concentrations ≤32.8 mg/dL had the highest hemorrhagic stroke risk compared to those with well-controlled blood pressure, LDL-C concentrations > 40.2 mg/dL, and aged ≤64.8 years (HR 41.7, 95%CI 17.2–101.6). Similar results were observed by excluding participants with eGFR < 60 ml/min/1.73 m^2^, 10-year Framingham risk score > 30%, BMI < 18.5 kg/m^2^, or who used blood pressure-lowering drugs or glucose-lowering drugs (Table 2).

**Fig. 3:**
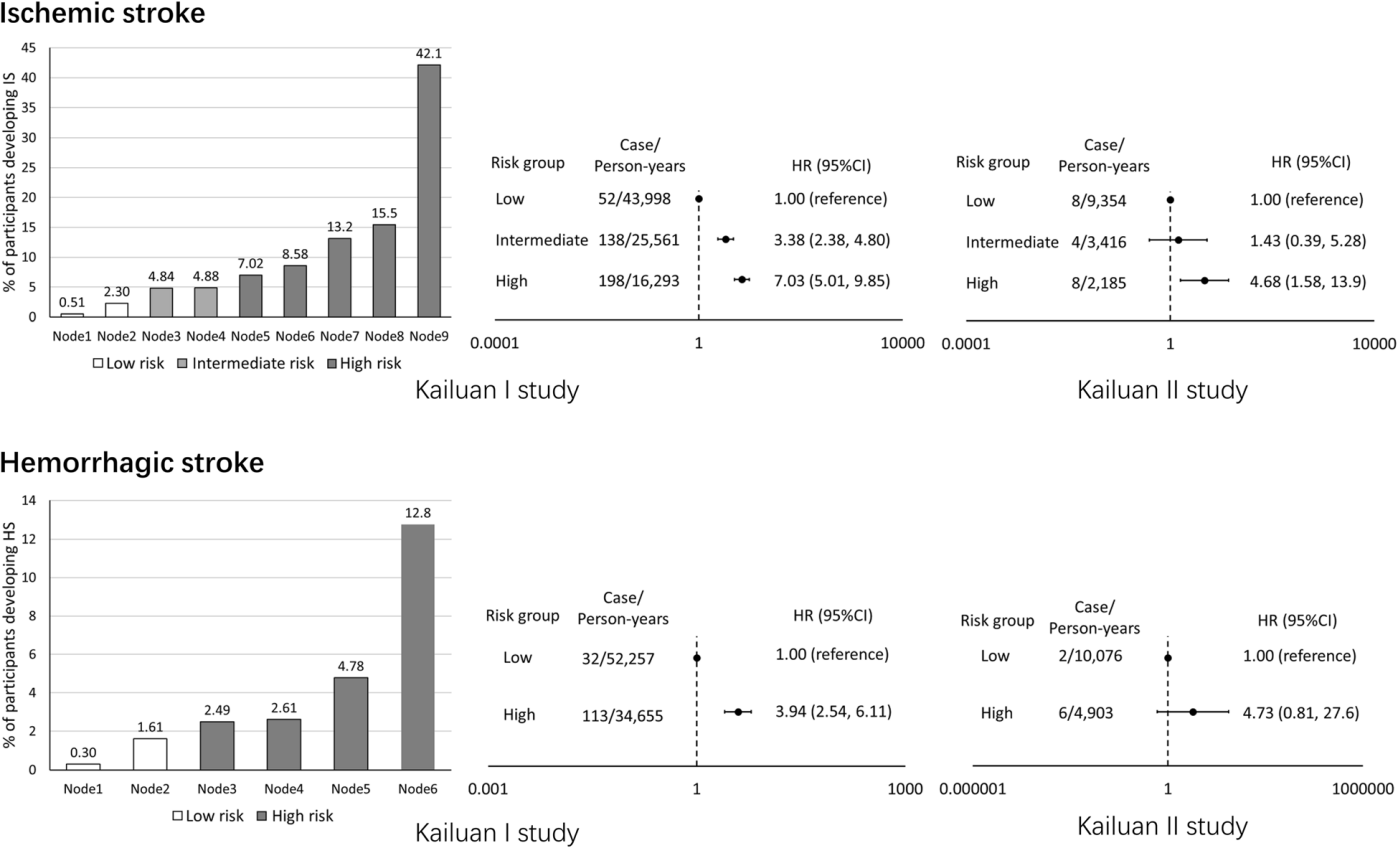
Percentages of participants who developed ischemic stroke and hemorrhagic stroke during follow-up and adjusted hazard ratios (HRs) and 95% confidence intervals (CIs) across the risk groups in individuals with low-density lipoprotein cholesterol concentrations < 70 mg/dL in the Kailuan I study and the Kailuan II study. IS, ischemic stroke; HS, hemorrhagic stroke

The Kailuan I study participants were stratified into high (> 5% developed ischemic stroke; n = 4548), intermediate (3–5% developed ischemic stroke; n = 2840), and low (< 3% developed ischemic stroke; n = 1939) risk groups. The ability of the derived risk tree to stratify participants into these groups was tested in the validation dataset (the Kailuan II study). A similar dose-response trend across the 3 risk groups for ischemic stroke was observed—the HRs for the high- versus low-risk groups were 7.03 (95%CI 5.01–9.85) in the training dataset and 4.68 (5%CI 1.58–13.9) in the validation dataset. The HRs for the high-risk group (> 2% developed hemorrhagic stroke, n = 5468) versus the low-risk group (< 2% developed hemorrhagic stroke, n = 3859) were 3.94 (95%CI 2.54–6.11) in the training dataset and 4.73 (5%CI 0.81–27.6) in the validation dataset (Fig. 3). The SCTREE model had a similar AUC and Brier score relative to the multivariate Cox model (Supplementary Table 3). The random survival forest analysis showed that blood pressure control and LDL-C concentrations were among the top predictors for both ischemic stroke and hemorrhagic stroke, and age was a strong predictor for ischemic stroke, which was consistent with the results of the SCTREE model (Supplementary Fig. 2). When we turned the outcomes into a classification one (stroke versus non-stroke event), the results did not materially change. Poorly controlled blood pressure and low LDL-C concentrations remained the top predictors of ischemic stroke and hemorrhagic stroke (Supplementary Fig. 3 & 4).

There was a linear association between cumulative average LDL-C concentrations and stroke risk. Very low LDL-C concentrations (< 40 mg/dL) were significantly associated with increased risk of ischemic stroke (HR 2.07, 95%CI 1.53, 2.80) and hemorrhagic stroke (HR 2.70, 95%CI 1.70, 4.30) compared to LDL-C concentrations of 55–70 mg/dL. These results were further confirmed using a quartile-based analysis (*P*_*trend*_ < 0.01 for both) (Table 3).

**Table 3:** Adjusted hazards ratios and 95% confidence intervals for ischemic stroke risk and hemorrhagic risk according to low-density lipoprotein cholesterol clinical cutoffs and quartiles in Kailuan I study participants with low-density lipoprotein cholesterol concentrations < 70 mg/dL

	Ischemic stroke					Hemorrhagic stroke				
	HR (95%CI)					HR (95%CI)				
LDL-C, mmol/L	≤40	40–55		55–70		≤40	40–55		55–70	
Number	982	2374		5971		982	2374		5971	
Case/person-years	90/8034	110/21,570		188/56,249		42/8341	34/21,846		69/56,725	
Mean LDL-C ± SD, mg/dL	26.7 ± 10.3	49.1 ± 4.05		63.0 ± 4.10		26.7 ± 10.3	49.1 ± 4.05		63.0 ± 4.10	
Multivariate model^a^	2.07 (1.53, 2.80)	1.43 (1.11, 1.84)		1.00 (reference)		2.70 (1.70, 4.30)	1.16 (0.74, 1.80)		1.00 (reference)	
Excluding eGFR < 60 ml/min/1.73 m^2^	2.21 (1.60, 3.07)	1.53 (1.17, 2.00)		1.00 (reference)		2.65 (1.63, 4.30)	1.13 (0.72, 1.77)		1.00 (reference)	
Excluding Framingham risk score > 30%	2.37 (1.57, 3.58)	1.48 (1.04, 2.10)		1.00 (reference)		2.60 (1.39, 4.86)	1.00 (0.53, 1.88)		1.00 (reference)	
Excluding BMI < 18.5 kg/m^2^	2.09 (1.54, 2.84)	1.45 (1.13, 1.87)		1.00 (reference)		2.69 (1.68, 4.31)	1.19 (0.76, 1.85)		1.00 (reference)	
Excluding blood pressure-lowering drugs	2.01 (1.41, 2.88)	1.40 (1.03, 1.91)		1.00 (reference)		2.56 (1.49, 4.40)	0.92 (0.53, 1.62)		1.00 (reference)	
Excluding glucose-lowering drugs	2.02 (1.48, 2.76)	1.45 (1.12, 1.88)		1.00 (reference)		2.70 (1.68, 4.33)	1.08 (0.69, 1.71)		1.00 (reference)	
**Quartiles of LDL-C**	**Q1**	**Q2**	**Q3**	**Q4**	***P***_***trend***_^**b**^	**Q1**	**Q2**	**Q3**	**Q4**	***P***_***trend***_
Number	2343	2333	2327	2324		2343	2333	2327	2324	
Case/person-years	153/20,356	108/21,562	72/21,893	55/22,040		60/20,800	40/21,824	23/22,088	22/22,199	
Mean LDL-C ± SD, mg/dL	38.1 ± 12.0	55.3 ± 2.43	62.1 ± 1.59	67.2 ± 1.38		38.1 ± 12.0	55.3 ± 2.43	62.1 ± 1.59	67.2 ± 1.38	
Multivariate model^a^	2.39 (1.69, 3.39)	2.40 (1.69, 3.41)	1.48 (1.02, 2.16)	1.00 (reference)	< 0.001	2.14 (1.24, 3.69)	1.89 (1.08, 3.31)	1.13 (0.61, 2.11)	1.00 (reference)	0.002
Excluding eGFR < 60 ml/min/1.73 m^2^	2.76 (1.89, 4.03)	2.68 (1.84, 3.92)	1.61 (1.07, 2.42)	1.00 (reference)	< 0.001	2.11 (1.20, 3.72)	1.87 (1.05, 3.32)	1.20 (0.64, 2.25)	1.00 (reference)	0.004
Excluding Framingham risk score > 30%	2.67 (1.63, 4.40)	2.62 (1.59, 4.30)	1.58 (0.93, 2.69)	1.00 (reference)	< 0.001	2.34 (1.07, 5.13)	1.99 (0.89, 4.46)	1.53 (0.66, 3.55)	1.00 (reference)	0.032
Excluding BMI < 18.5 kg/m^2^	2.40 (1.70, 3.41)	2.41 (1.70, 3.42)	1.47 (1.01, 2.15)	1.00 (reference)	< 0.001	2.13 (1.23, 3.68)	1.92 (1.10, 3.37)	1.09 (0.58, 2.05)	1.00 (reference)	0.002
Excluding blood pressure-lowering drugs	2.38 (1.56, 3.63)	2.32 (1.51, 3.55)	1.53 (0.97, 2.41)	1.00 (reference)	< 0.001	2.01 (1.04, 3.87)	1.68 (0.85, 3.33)	1.05 (0.49, 2.23)	1.00 (reference)	0.013
Excluding glucose-lowering drugs	2.37 (1.65, 3.39)	2.36 (1.64, 3.38)	1.39 (0.94, 2.05)	1.00 (reference)	< 0.001	2.15 (1.23, 3.75)	1.79 (1.00, 3.19)	1.18 (0.63, 2.21)	1.00 (reference)	0.002

*Abbreviations*: *HR*, hazard ratio; *CI*, confidence interval; *LDL-C*, low-density lipoprotein cholesterol; *SD*, standard deviation; *eGFR*, estimated glomerular filtration rate; *BMI*, body mass index

^a^All models were adjusted for sex (men or women), age (year), physical activity (inactive, moderately active, or vigorously active), smoking and drinking status (never, former, occasional or daily), blood pressure status during follow-up (well-controlled or poorly controlled), blood glucose status during follow-up (well-controlled or poorly controlled), body mass index (kg/m^2^), urine protein (negative, trace, +, ++, or +++), heart rate (bpm), triglyceride (mg/dL), high-density lipoprotein cholesterol (mg/dL), low-density lipoprotein cholesterol (mg/dL), estimated glomerular filtration rate (ml/min/1.73 m^2^), and high-sensitivity C-reactive protein (mg/L)

^b^Test for trend according to the variable containing the median value of each quartile

## Discussion

Using data from 2 community-based cohorts and a machine learning approach, we found that in participants with LDL-C concentrations< 70 mg/dL, and not receiving lipid-lowering therapy, the major attributes of stroke risk were very low LDL-C concentrations and poorly controlled blood pressure. The highest risk group, characterized by the presence of 2–3 of these risk factors, was at high risk of developing stroke during the 8.5- to 9-year follow-up period relative to the lowest risk group when predicted using either the training or the validation datasets. There was remarkable consistency between the two datasets. We further confirmed the association between low LDL-C concentrations and stroke risk using the traditional Cox proportional hazards model. For the primary prevention of stroke, these findings highlight the need for a better understanding of the influence of potential confounders in individuals with very low LDL-C concentrations in the absence of therapy.

The predictive models indicated that individuals with very low LDL-C concentrations without the influence of lipid-lowing drugs were still at elevated risk for stroke. One possible interpretation of these findings is that a very low LDL-C concentration is a marker of a chronic metabolic disorder and associated adverse sequelae of the disorder such as high inflammatory burden. Systemic chronic inflammation may lead to very low blood LDL-C concentrations by exacerbating cholesterol accumulation into macrophages [32]. Therefore, non-treatment and on-treatment low LDL-C concentrations may have different associations with cerebrovascular disease. Whether the observed results could apply to intervention trials remains to be elucidated.

Our study suggested the appropriate concentration of LDL-C below which stroke events identified was 33 mg/dL. Interestingly, this level is similar to the neonatal LDL-C concentrations at birth (21–39 mg/dL) [33]. Cholesterol is a constituent of cell membranes, hence, essential to maintain cellular structural integrity and serves as a precursor for bioactive compounds, ranging from steroid hormones to vitamin D. Plasma LDL-C concentrations of 21–39 mg/dL have been suggested to be the lower limit that will sustain normal cellular function [34–36]. In the Reasons for Geographical and Racial Differences in Stroke (REGARDS) study, participants with high LDL-C (≥70 mg/dL) and low hs-CRP (< 2 mg/L) had a lower risk of stroke [37]. A recent randomized trial reported that high-dose atorvastatin significantly reduced the overall incidence of stroke and CVD but increased the risk of hemorrhagic stroke [38]. Three recent large-scale observational studies reported that LDL-C concentrations < 70 mg/dL were positively associated with hemorrhagic stroke risk [8, 9]. The causal relevance of these observed associations between low LDL-C concentrations and hemorrhagic stroke was confirmed in a meta-analysis of LDL-C-lowering intervention and a Mendelian randomization analysis [39]. Interestingly, another Mendelian randomization analysis reported that decrement of the LDL-C concentrations may lead to decreased CVD risk but increased DM risk [40].

Of note, some guidelines [2, 41], although not consistently [27], comment on the potential adverse effect of very low LDL-C concentrations, in the range of 25 to 70 mg/dL, achieved with lipid-lowering therapy. The recent 2019 European Society of Cardiology/European Atherosclerosis Society lipid guidelines recommended a lower LDL-C goal (e.g., < 55 mg/dL) than the previous guidelines for individuals at very-high CVD risk [27]. The authors of the guidelines indicated there are no known adverse effects of LDL-C concentrations < 40 mg/dL [27].

Our result suggested poorly controlled blood pressure contributed to the risk of stroke in individuals with very low LDL-C concentrations. Poorly controlled blood pressure or glucose conferred 1.5–2-fold increased risk of stroke [42]. Individuals of Asian descent have a higher prevalence of metabolic syndrome than of Caucasians [41]. Solely increment in lowering LDL-C is not as effective in reducing atherosclerotic risk more in Asians compared to Caucasians. The Evaluation of Cardiovascular Outcomes After an Acute Coronary Syndrome During Treatment with Alirocumab study demonstrated that LDL-C lowering with alirocumab significantly reduced the primary CVD outcomes in North Americans (HR 0.78, 95%CI 0.65–0.94), but not in Asians (HR 1.03, 95%CI 0.76–1.38) [43].

Our study has several strengths, including its large sample size of participants with LDL-C concentrations < 70 mg/dL. The SCTREE analysis, beyond the traditional statistical analyses, provides a robust framework for testing attributes that are predictive of stroke risk taking the complex high-order interactions into account. We excluded people using lipid-modifying drugs to reduce the sources of potential confounding related to these medications. The ability to use cumulative average values for all continuous variables in the SCTREE model reduced the possibility of “regression dilution.”

Our study has several limitations. Our study is based on two Chinese cohorts, which limits the generalizability of our findings to other ethnic groups. Further, the mean age of the validation cohort (43.6 years) was much lower than that of the training cohort (57.3 years), which implied that a smaller proportion of high-risk participants was included in the validation analysis. The sample size of our study (n = 9327) was relatively small because of our strict inclusion criteria, and we thus identified a small number of incident ischemic stroke events (n = 388) and hemorrhagic stroke events (n = 145) during follow-up, which limited the detection of some potential weak-to-moderate predictors because of inadequate statistical power in each terminal node, the stopping rules or the competitive importance of the variables/pruning procedure. The small number of incident ischemic stroke events (n = 20) and hemorrhagic stroke events (n = 8) limited the detection of significant predictive combinations in the validation set, and we could not swap the train and validation set to confirm the results. However, similar associations were observed in both cohorts. We did not measure hemoglobin A1c because of its high cost as a screening test in the general population, and some individuals with poorly controlled blood glucose could be misclassified. The proportion of hemorrhagic stroke in our cohort with LDL-C concentrations < 70 mg/dL was higher than that in the general population [18] because the ischemic stroke events attributed to high LDL-C concentrations were excluded. Further study or publicly datasets with repeated LDL-C concentrations may help to validate our results.

## Conclusions

In a Chinese population with LDL-C concentrations < 70 mg/dL, very low concentrations of LDL-C, incorporating poorly controlled blood pressure, and older age significantly predicted the occurrence of ischemic stroke and hemorrhagic stroke. Additional data are required to confirm our findings in a population with different ethnic and social-economic backgrounds.

## Supplementary Information

**Additional file 1.** Table S1. Explanatory notes for all clinical characteristics included in the survival conditional inference tree model. Table S2. The risk of ischemic stroke and hemorrhagic stroke, according to the status of top three predictors, identified by the survival conditional inference tree model, in the Kailuan I study participants with low density lipoprotein cholesterol concentrations<70mg/dL. Table S3. Area under receiver operating characteristic curves and brier score for ischemic stroke and hemorrhagic stroke in Kailuan I study participants with low density lipoprotein cholesterol concentrations<70mg/dL. Fig. S1. Flow charts showing the selection strategy of individuals with low density lipoprotein cholesterol concentrations < 70mg/dL in the Kailuan I study and Kailuan II study. Fig. S2. The variable importance of all predictors from the random survival forest analysis for ischemic stroke and hemorrhagic stroke. Fig. S3. Conditional inference tree for ischemic stroke vs non-ischemic stroke in individuals with baseline and cumulative average low density lipoprotein cholesterol concentrations < 70 mg/dL. Fig. S4. Conditional inference tree for hemorrhagic stroke vs non-hemorrhagic stroke in individuals with baseline and cumulative average low density lipoprotein cholesterol concentrations < 70 mg/dL.

## Data Availability

The dataset analyzed during the current study is not publicly available since the consent obtained from the participants did not include permission for their data to be shared publicly. However, the data are available from the principal investigator (Shouling Wu) on reasonable request.

## Abbreviations

CI: Confidence interval
CVD: Cardiovascular diseases
HR: Hazard ratio
ICD: International Classification of Diseases
LDL-C: Low-density lipoprotein cholesterol
SCTREE: Survival conditional inference tree
