# Supplementary material for: The risk of ischemic stroke and hemorrhagic stroke in Chinese adults with low-density lipoprotein cholesterol concentrations < 70 mg/dL
Source: BMC Med. 2021 Jun 16;19:142. doi: 10.1186/s12916-021-02014-4 (PMC8207613; doi:10.1186/s12916-021-02014-4)
Supplement: Supplementary file 1 — Additional file 1. Table S1. Explanatory notes for all clinical characteristics included in the survival conditional inference tree model. Table S2. The risk of ischemic stroke and hemorrhagic stroke, according to the status of top three predictors, identified by the survival conditional inference tree model, in the Kailuan I study participants with low density lipoprotein cholesterol concentrations<70mg/dL. Table S3. Area under receiver operating characteristic curves and brier score for ischemic stroke and hemorrhagic stroke in Kailuan I study participants with low density lipoprotein cholesterol concentrations<70mg/dL. Fig. S1. Flow charts showing the selection strategy of individuals with low density lipoprotein cholesterol concentrations < 70mg/dL in the Kailuan I study and Kailuan II study. Fig. S2. The variable importance of all predictors from the random survival forest analysis for ischemic stroke and hemorrhagic stroke. Fig. S3. Conditional inference tree for ischemic stroke vs non-ischemic stroke in individuals with baseline and cumulative average low density lipoprotein cholesterol concentrations < 70 mg/dL. Fig. S4. Conditional inference tree for hemorrhagic stroke vs non-hemorrhagic stroke in individuals with baseline and cumulative average low density lipoprotein cholesterol concentrations < 70 mg/dL. [file 12916_2021_2014_MOESM1_ESM.docx]

**Supplementary material**

**The risk of ischemic stroke and hemorrhagic stroke in Chinese adults with low density lipoprotein cholesterol concentrations < 70 mg/dL**

Zhijun Wu^1,+^, Zhe Huang^2,+^, Alice H. Lichtenstein^3^, Yesong Liu^4^, Shuohua Chen^5^, Yao Jin^1^, Muzi Na^6^, Le Bao^7^, Shouling Wu^2^ & Xiang Gao^6^

From the ^1^ Department of Cardiology, Ruijin Hospital, Shanghai Jiaotong University School of Medicine, Shanghai, People’s Republic of China; ^2^ Department of Cardiology, Kailuan General Hospital, Tangshan, People’s Republic of China; ^3^ Cardiovascular Nutrition Laboratory, JM USDA Human Nutrition Research Center on Aging, Tufts University, Boston, MA ; ^4^ Department of Neurology, Kailuan General Hospital, Tangshan, People’s Republic of China; ^5^ Health Care Center, Kailuan Medical Group, Tangshan, People’s Republic of China; ^6^ Department of Nutritional Sciences, Pennsylvania State University, State college, PA ; and ^7^ Department of Statistics, Pennsylvania State University, State college, PA

^+^Contribution equally.

**Table S1. Explanatory notes for all clinical characteristics included in the survival conditional inference tree model**

| Characteristics | Explanatory notes |
| --- | --- |
| Age (year) | Age at recruitment |
| Sex (0,1) | 0=Female, 1=Male |
| Physical activity (1-3) | Self-reported. 1= Inactive, 2=Moderately active, 3=Vigorously active |
| Smoking status (1-4) | Self-reported. 1=Never, 2=Former, 3=Occasional, 4=Daily |
| Alcohol consumption (1-4) | Self-reported. 1=Never, 2=Former, 3=Occasional, 4=Daily |
| Blood pressure control (1-4) | Self-reported and blood pressure measured at baseline and during follow-up. 1= SBP < 140mmHg and DBP < 90mmHg without treatment, 2= SBP < 140mmHg and DBP < 90mmHg after treatment, 3= SBP ≥ 140mmHg or DBP ≥ 90mmHg without treatment, 4= SBP ≥ 140mmHg or DBP ≥ 90mmHg after treatment |
| Blood glucose control (1-4) | Self-reported and blood glucose measured at baseline and during follow-up. 1= FBG < 126mg/dL without treatment, 2= FBG < 126mg/dL after treatment, 3= FBG ≥ 126mg/dL without treatment, 4= FBG ≥ 126mg/dL after treatment |
| Urine protein (0-4) | Urine test at baseline and during follow-up. 0=Negative, 1=Trace, 2=+, 3=++, 4=+++ |
| BMI (kg/m^2^) | Measured at baseline and during follow-up. Cumulative average values used. |
| TG (mg/dL) | Lipid test at baseline and during follow-up. Cumulative average values used. |
| HR (bpm) | Tested at baseline and during follow-up. Cumulative average values used. |
| LDL-C (mg/dL) | Lipid test at baseline and during follow-up. Cumulative average values used. |
| HDL-C (mg/dL) | Lipid test at baseline and during follow-up. Cumulative average values used. |
| eGFR, ml/min/1.73m^2^ | Blood test at baseline and during follow-up. Cumulative average values used. |
| hs-CRP, mg/L | Blood test at baseline and during follow-up. Cumulative average values used. |

Abbreviations: SBP, systolic blood pressure; DBP, diastolic blood pressure; FBG, fasting blood glucose; BMI, body mass index; TG, triglyceride; HR: heart rate; LDL-C, low density lipoprotein cholesterol; HDL-C, high density lipoprotein cholesterol; eGFR, estimated glomerular filtration rate; hs-CRP, high sensitivity C-reactive protein.

**Table S2. The risk of** **ischemic stroke and hemorrhagic stroke, according to the status of top three predictors, identified by the survival conditional inference tree model, in the Kailuan I study participants with low density lipoprotein cholesterol concentrations<70mg/dL.**

| Attributes | Cut-off^a^ | HR (95%CI)^b^ | | |
| --- | --- | --- | --- | --- |
|  |  | Case number/ person-years | Cox | PSM-Cox |
| **Ischemic stroke** | | | | |
| LDL-C, mg/dL | > 33.2 | 312/80,749 | 1.00 (reference) | 1.00 (reference) |
|  | ≤ 33.2 | 76/5,104 | 2.19 (1.59, 3.01) | 2.61 (1.52, 4.50) |
| Blood pressure | Well-controlled | 130/56,391 | 1.00 (reference) | 1.00 (reference) |
|  | Poorly-controlled | 258/29,462 | 2.48 (1.94, 3.18) | 2.16 (1.66, 2.81) |
| Age, year | ≤ 64.9 | 193/62,748 | 1.00 (reference) | 1.00 (reference) |
|  | > 64.9 | 195/23,105 | 1.55 (1.20, 2.01) | 1.80 (1.05, 3.08) |
| **Hemorrhagic stroke** | | | | |
| LDL-C, mg/dL | > 32.8 | 115/81,661 | 1.00 (reference) | 1.00 (reference) |
|  | ≤ 32.8 | 30/5,251 | 2.24 (1.33, 3.76) | 4.97 (1.72, 14.4) |
| Blood pressure | Well-controlled | 45/56,692 | 1.00 (reference) | 1.00 (reference) |
|  | Poorly-controlled | 100/30,220 | 3.13 (2.06, 4.76) | 2.76 (1.77, 4.33) |

Abbreviations: CVD, cardiovascular disease; HR, hazard ratio; CI, confidence interval; LDL-C, low density lipoprotein cholesterol; PSM, propensity score matched; HTN, hypertension; DM, diabetes mellitus.

^a^ The cut-off points were identified by the survival conditional inference tree model.

^b^ All models were adjusted for sex (men or women), age (year), physical activity (inactive, moderately active or vigorously active), smoking and drinking status (never, former, occasional or daily), blood pressure status during follow-up (well-controlled or poorly-controlled), blood glucose status during follow-up (well-controlled or poorly-controlled), body mass index (kg/m^2^), urine protein (negative, trace, +, ++ or +++), heart rate (bpm), triglyceride (mg/dL), high density lipoprotein cholesterol (mg/dL), low density lipoprotein cholesterol (mg/dL), estimated glomerular filtration rate (ml/min/1.73m^2^) and high sensitivity C-reactive protein (mg/L).

**Table S3. Area under receiver operating characteristic curves and brier score for ischemic stroke and hemorrhagic stroke in Kailuan I study participants with low density lipoprotein cholesterol concentrations<70mg/dL.**

| Model | Ischemic stroke | Hemorrhagic stroke |
| --- | --- | --- |
| **Area under receiver operating characteristic curves, %** | | |
| SCTREE | 76.7 (74.4, 79.1) | 79.6 (76.3, 82.9) |
| COX ^a^ | 76.9 (74.7, 79.2) | 78.9 (75.4, 82.4) |
| **Brier score** | | |
| SCTREE | 0.037 (0.002, 0.040) | 0.014 (0.012, 0.017) |
| COX | 0.037 (0.033, 0.041) | 0.014 (0.012, 0.017) |

Abbreviations: SCTREE: survival conditional inference tree

^a^ All models were adjusted for sex (men or women), age (year), physical activity (inactive, moderately active or vigorously active), smoking and drinking status (never, former, occasional or daily), blood pressure status during follow-up (well-controlled or poorly-controlled), blood glucose status during follow-up (well-controlled or poorly-controlled), body mass index (kg/m^2^), urine protein (negative, trace, +, ++ or +++), heart rate (bpm), triglyceride (mg/dL), high density lipoprotein cholesterol (mg/dL), low density lipoprotein cholesterol (mg/dL), estimated glomerular filtration rate (ml/min/1.73m^2^) and high sensitivity C-reactive protein (mg/L).


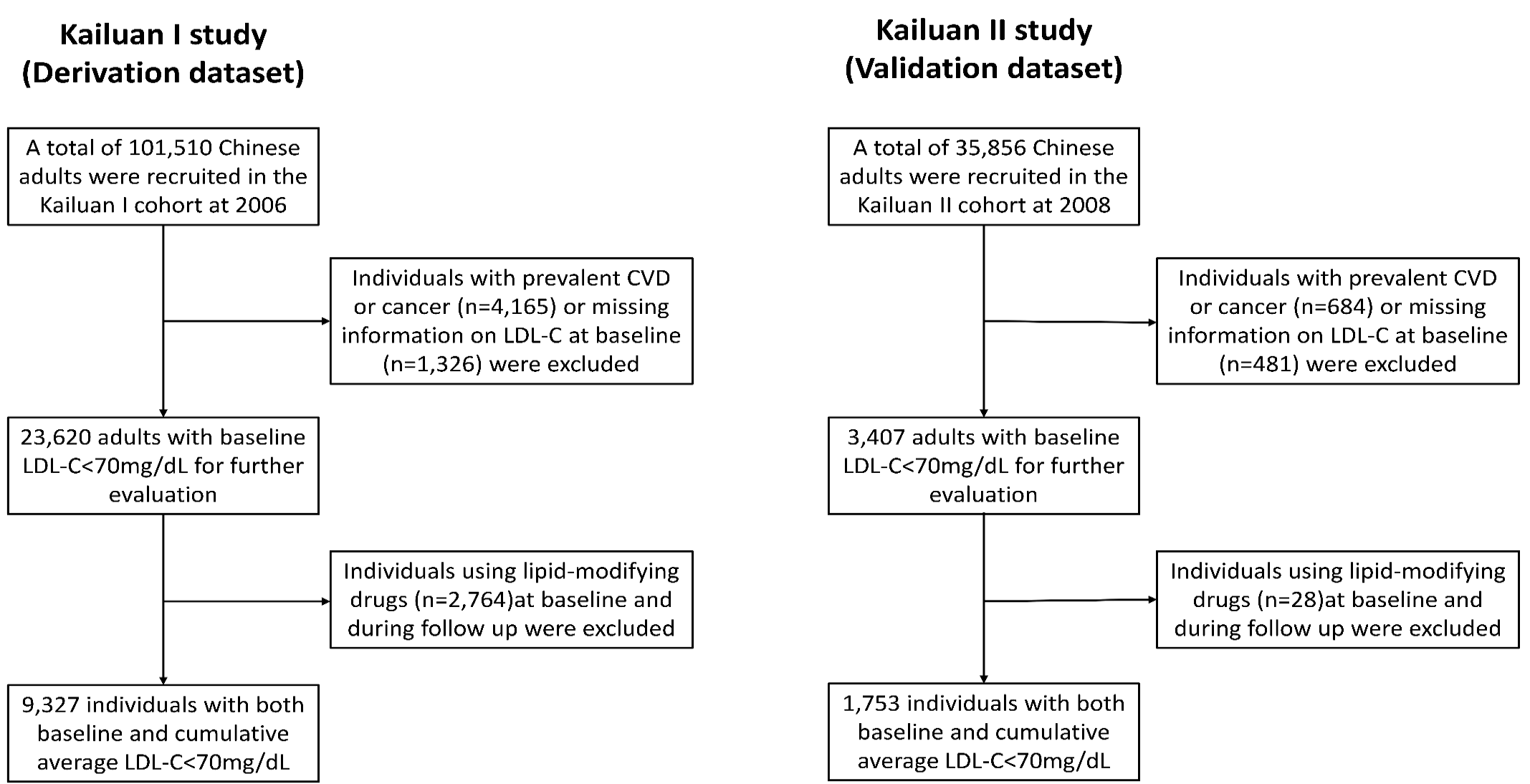


**Fig.S1. Flow charts showing the selection strategy of individuals with low density lipoprotein cholesterol concentrations < 70mg/dL in the Kailuan I study and Kailuan II study**. CVD, cardiovascular disease; LDL-C, low density lipoprotein cholesterol.


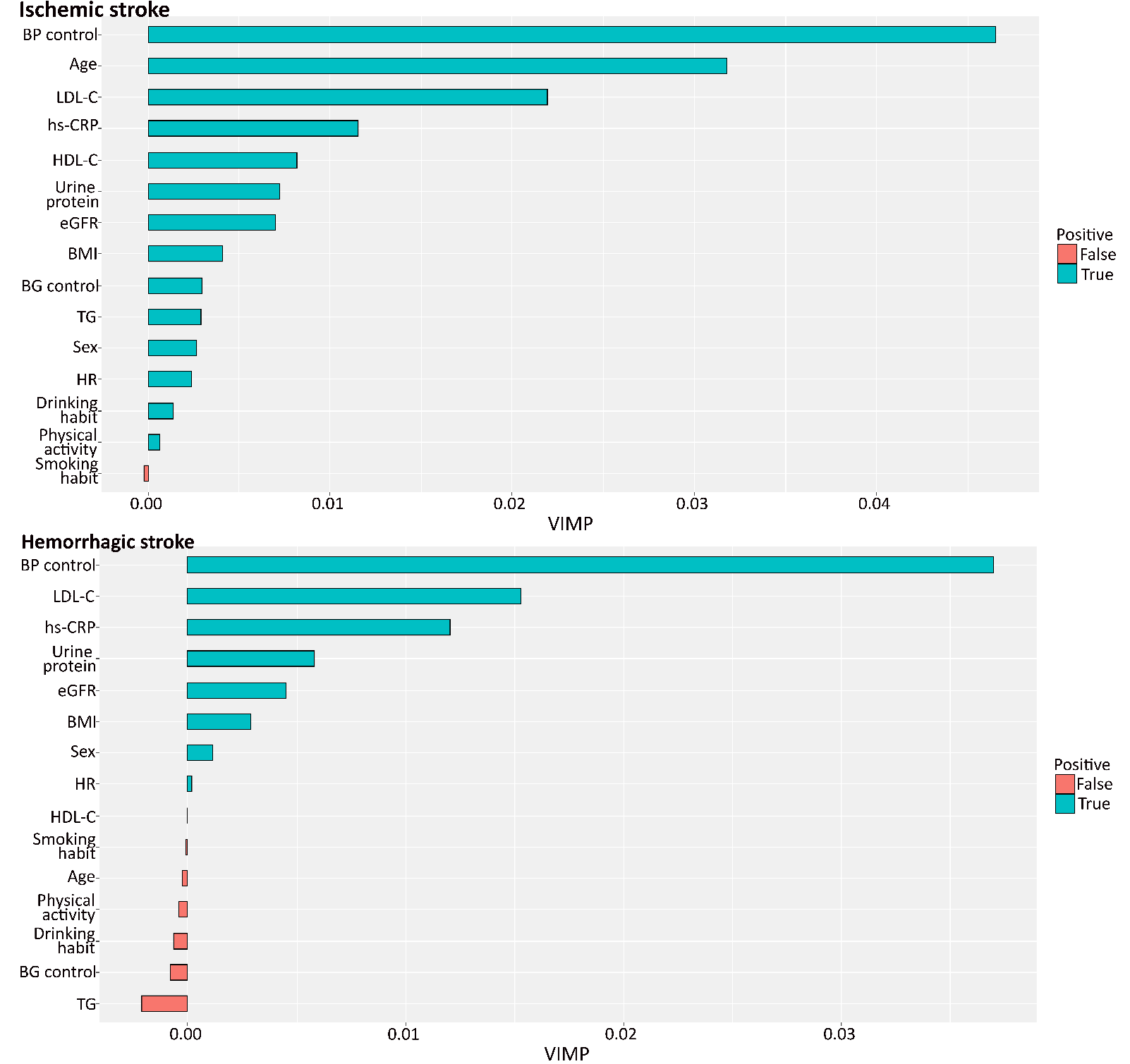


**Fig.S2. The variable importance of all predictors from the random survival forest analysis for ischemic stroke and hemorrhagic stroke.** BP, blood pressure; LDL-C, low density lipoprotein cholesterol; hs-CRP, high sensitivity C-reactive protein; HDL-C, high density lipoprotein cholesterol; eGFR, estimated glomerular filtration rate; BMI, body mass index; BG, blood glucose; TG, triglyceride; HR: heart rate; VIMP, variable importance measure.


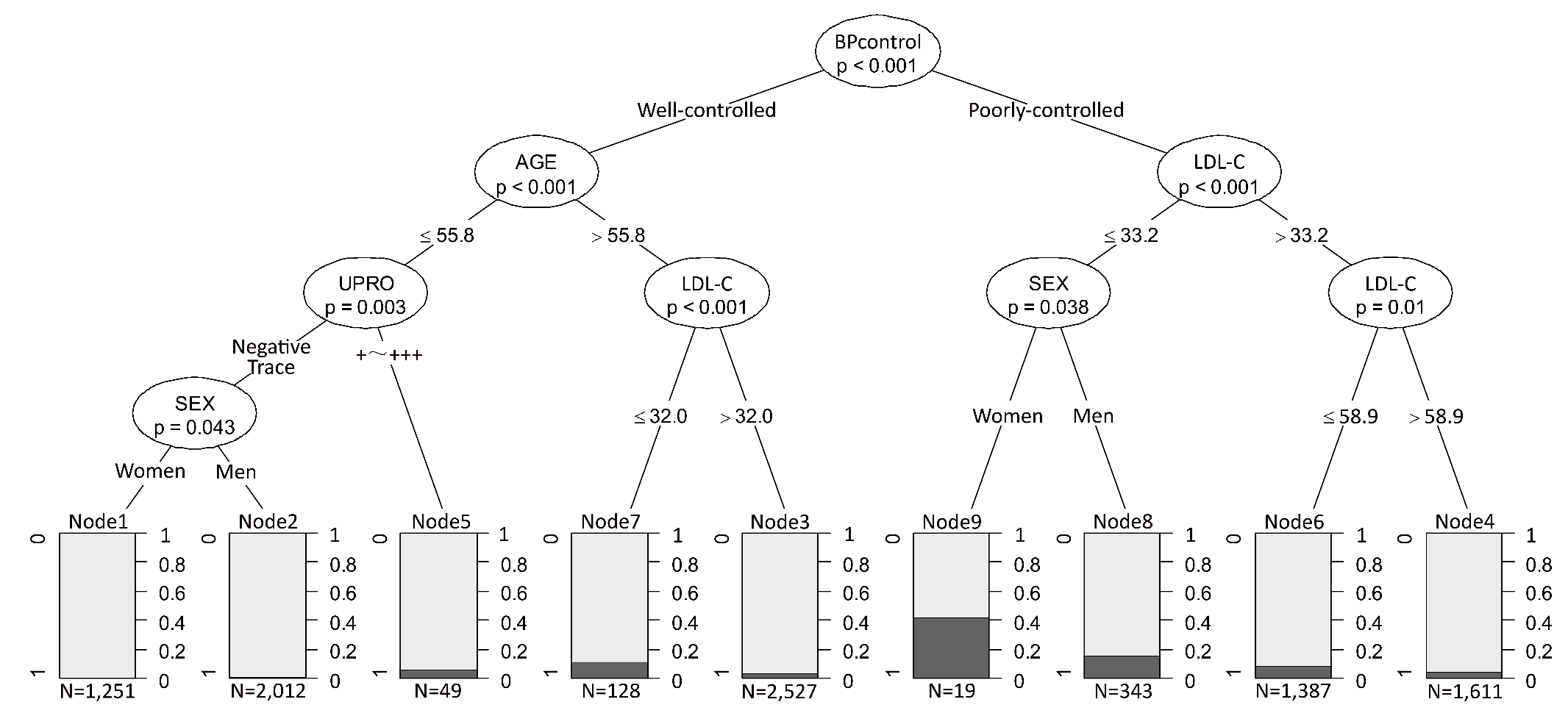


**Fig.S3.** **Conditional inference tree for ischemic stroke vs non-ischemic stroke in individuals with baseline and cumulative average low density lipoprotein cholesterol concentrations < 70 mg/dL.** The terminal nodes show the proportion of ischemic stroke events. BP, blood pressure; LDL-C, low density lipoprotein cholesterol; UPRO, urine protein.


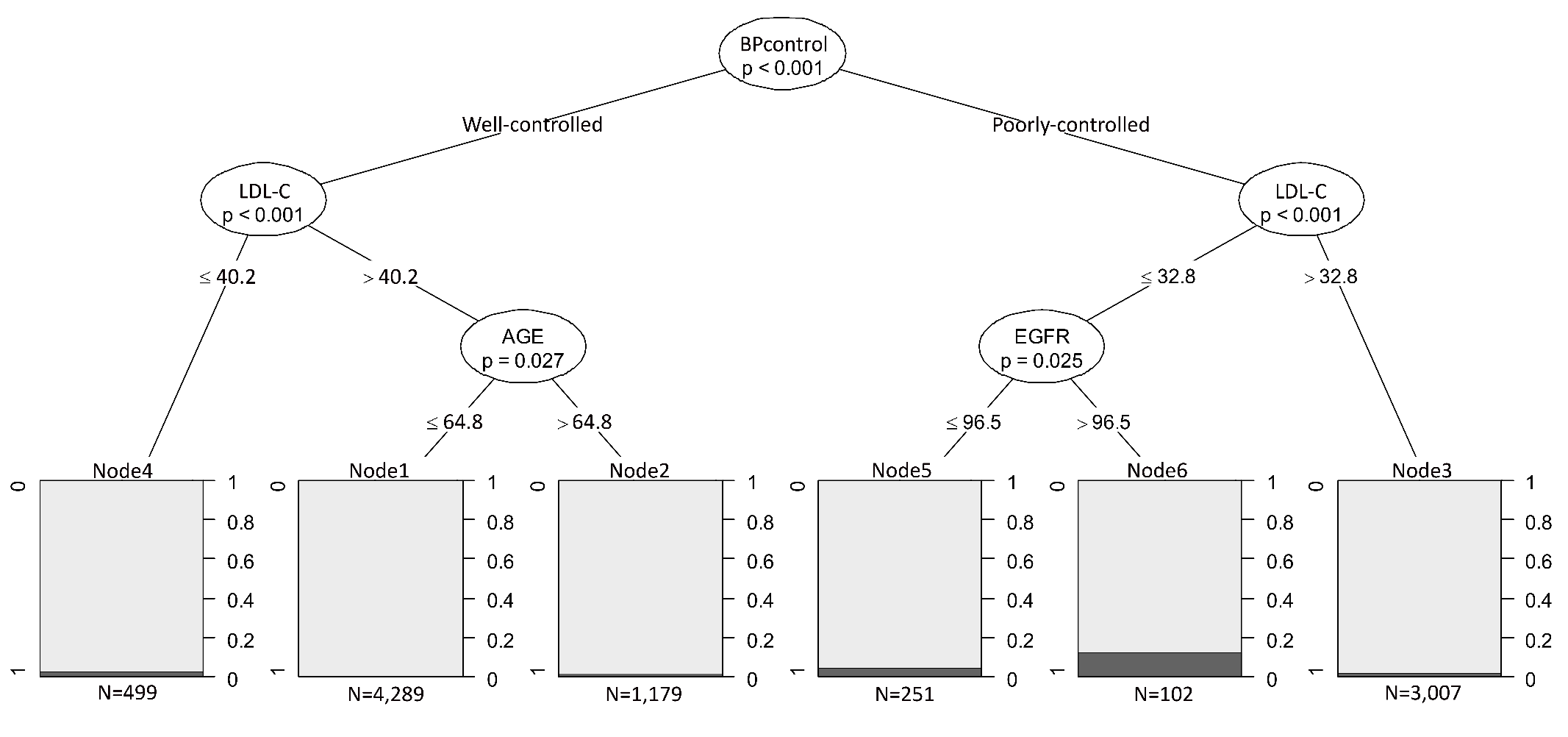


**Fig.S4.** **Conditional inference tree for hemorrhagic stroke vs non-hemorrhagic stroke in individuals with baseline and cumulative average low density lipoprotein cholesterol concentrations < 70 mg/dL.** The terminal nodes show the proportion of hemorrhagic stroke events. BP, blood pressure; LDL-C, low density lipoprotein cholesterol; EGFR, estimated glomerular filtration rate.
